# Aging and Veterinary Care of Cats, Dogs, and Horses through the Records of Three University Veterinary Hospitals

**DOI:** 10.3389/fvets.2017.00014

**Published:** 2017-02-14

**Authors:** Bruno Cozzi, Cristina Ballarin, Roberto Mantovani, Ada Rota

**Affiliations:** ^1^Department of Comparative Biomedicine and Food Science, University of Padova, Padova, Italy; ^2^Department of Agronomy, Food, Natural resources, Animals and Environment, University of Padova, Padova, Italy; ^3^Department of Veterinary Sciences, University of Turin, Turin, Italy

**Keywords:** animal life span, aging, animal gerontology, dog, cat, horse, animal hospital

## Abstract

The present article examines over 63,000 medical records belonging to the Veterinary Hospitals of the Universities of Bologna, Torino, and Padova, all in Northern Italy, and relative to dogs (approximately 50,000), cats (approximately 12,000), and companion horses (slightly less than 1,000). The animals of the three species were divided into age classes and categorized per sex into males, females, and neutered individuals. The mean age at visit and the effects of age classes and category (analyzed *via* ANOVA) are presented and discussed. The data indicate that many animals are presented to the hospitals either in the early phases of their life (presumably for vaccination and, in cats and dogs, gonadectomy) or in the advanced age (over 10 years in dogs, over 15 years in cats, and over 17 years in horses). The records of very old individuals of the three species are also reported. On the whole, the results suggest that a growing population of mature to old domestic carnivores or companion horses reaches ages that were considered exceptional only a few years ago. The data also testify an evolution in the animal–owner relationship and a renewed respect for the value of life in companion domestic mammals.

## Introduction

Human life expectancy increased tremendously in the industrial society during the twentieth and twenty-first centuries for a number of reasons connected to satisfactory nutrition (including water availability), improved health care (including mass vaccination), proper housing conditions (including heating), and the relative absence of global conflicts after World War II (1).

As a result of the improved life conditions, an ever-growing part of the population of North America, Europe, Australia, Japan, Russia, and other prosperous countries reaches older age (>80 years old) and prompts the flourishing of the modern science of health for the elderly (2). The general aging of the human population brings new social perceptions, adds cultural developments to the contemporary societies, and obviously implies new costs (3, 4).

Although the debate on the lifespan and the theories of aging have been centered mostly on humans [for general reference, see Ref. (5–7)], several specialized articles have also discussed longevity in animal species [Ref. (8–18); for a comparative overview, see Ref (19)]. However, the majority of these investigations were directed mostly at the observation of the maximum longevity records available for mammals and other vertebrates. In fact, the difference between absolute record (the oldest recorded living individual of a given species) and the potential lifespan (how much an individual of a given species may expect to live) is huge and requires additional consideration on the representativeness of the observed cohort, the trustfulness of the records, and the statistical methods employed.

In the present world, companion animals, and especially dogs and cats, have acquired a relevant role in social habits and are a very diffuse presence in urban households or countryside. Based on the emotional attachment of their owners, companion species receive dedicated attention and qualified veterinary care. The horse, one of the oldest domesticated species, is bred either as a companion animal (including also horses used in sport activities) or for meat production (at least in some countries where horse meat is a legal and popular human food or in developing countries). Whenever horses are bred as companion animals, their situation is comparable to that of domestic carnivores, even with all the obvious differences of the costs of daily maintenance and the required space. So cats, dogs, and companion horses may be considered as living beings that share the daily environment with humans, receive proper food prepared under controlled hygienic conditions, and are monitored by veterinary specialists that strive to reach quality levels somehow comparable to those reserved to humans, including, at least in part, the specific health care dedicated to the human elderly.

Following the abovementioned considerations, the present study, deals with aging of domestic carnivores and companion horses evaluating the progressive age classes at visit, using the databases of three relevant veterinary university hospitals of Northern Italy. The extent of their life expectancy is discussed also in reference to what already known from past studies, considering also information pertinent to other key species and the general context of a modern industrial society.

## Materials and Methods

### Animal Data

For the present study, we used the databases of the Veterinary Hospitals of the Universities of Torino, Padova, and Bologna (see Table 1), all located in Northern Italy.

**Table 1:** **Data from the veterinary medical hospitals of Torino, Padova, and Bologna (Italy)**.

Hospital	Years	# of cases	# of dogs	# of cats	# of horses
Torino	2009–2015	24,410	18,470	5,459	481
Padova	2010–2015	25,635	21,439	3,942	254
Bologna	2011–2015	13,128	9,457	3,452	219
Total		63,173	49,366	12,853	954

*Table reports a synthesis of these data collected in the three reference veterinary hospitals, subdivided by years and species. Full data are available as supplementary material*.

Sensible data [owner’s identification (id), name of the visiting physician] were removed from the files, leaving only species, sex, and age. Data relative to animal id and breed were often incomplete. Based on the Italian law, horses must be classified at birth as either companion horses (including sport horses) or horses bred for commercial purposes (meat production). The formers cannot be slaughtered for meat consumption, and their lifespan is accordingly consistently longer. On the other hand, horses bred for meat production were not considered in the present study because they are generally slaughtered at a young age.

The original databases included also data relative to clinical examinations of bovine, goats, sheep, pigs, ferrets, rabbits, and other less common mammals. A certain number of visits concerned also birds and reptiles (snakes and iguanas mostly). However, we excluded data on ruminants, pigs, and the other less frequent species because they were incomplete or fragmentary. Furthermore, the lifespan of species intended for meat or milk production is limited by the industrial process of their production, and, therefore, of reduced interests for the scope of the present study.

Prior to utilization, the data were combed for anomalies and inconsistencies. When the precise date of birth was unknown, we calculated the age of the animal by considering only the year of birth, whenever available.

Extra care was taken to verify the reliability of these data relative to very old animals of the three species.

### Age Classes and Criteria for Subdivision

The examined dogs, cats, and companion horses were subdivided into age classes. For carnivores, since the onset of puberty usually takes place before the end of the first year and a category inclusive of prepubertal individuals only would have spanned a very short interval, we decided to include in the first age class very young animals from birth to less than 2 years of age, thus including prepubertal and early pubertal individuals. The first age class of companion and sport horses includes pre-pubertal colts of less than 3 years of age. We then considered as much as possible homogeneous intervals accounting for different life phases. In cats, the subsequent phases were considered as follows: young (2–4 years), young adult (5–7 years), adult (8–10 years), middle aged (11–15 years), young senior (16–18 years), and senior (>19 years). However, in dogs, the lower number of animals aged >16 years suggested a unique final class of senior (>16 years). In companion horses, the age classes were defined on a different basis, considering the different life pattern (before and after puberty) and the sample size. Age classes for companion horses after puberty were then classified as follows: young (3–4 years), adult (5–11 years), middle-aged young senior (12–16 years), and senior (>17 years).

### Statistical Methods

At first, a unique database containing 63,173 observations (49,366 for dogs, 12,853 for cats, and 954 for horses) was created reporting the hospital, the species, the examination year, and the individual sex and age at the visit. After classification of the age class as described above, a Chi-Square goodness-of-fit test was carried out to test the hypotheses, within each hospital, of a homogeneous distribution of different age classes in different examination years.

A further one-way ANOVA on age (in years) as affected by sexes was performed accounting for four different sexes in dogs and cats (i.e., males, M; neutered males, C; females, F; and spayed females, S) and three sexes in companion horses (M, C, and F). The hypothesis test for the significance of the age effect was based on a one-tailed *F* statistics. Multiple comparison tests between least square means (LSMEANS) were carried by adjusting the probability of mean comparison by the Bonferroni methods. Contrasts between intact vs. neutered animals or intact and neutered individuals of the same sex were also carried out for dogs and cats species. A *p* ≤ 0.05 was chosen as threshold for statistically significant results.

## Results

Raw data were examined and the results for each species divided into age classes and first analyzed independently for each hospital. The frequency (percentage) of visits relative to age in the three hospitals is reported below for the single species.

### Dogs

**Figure 1:**
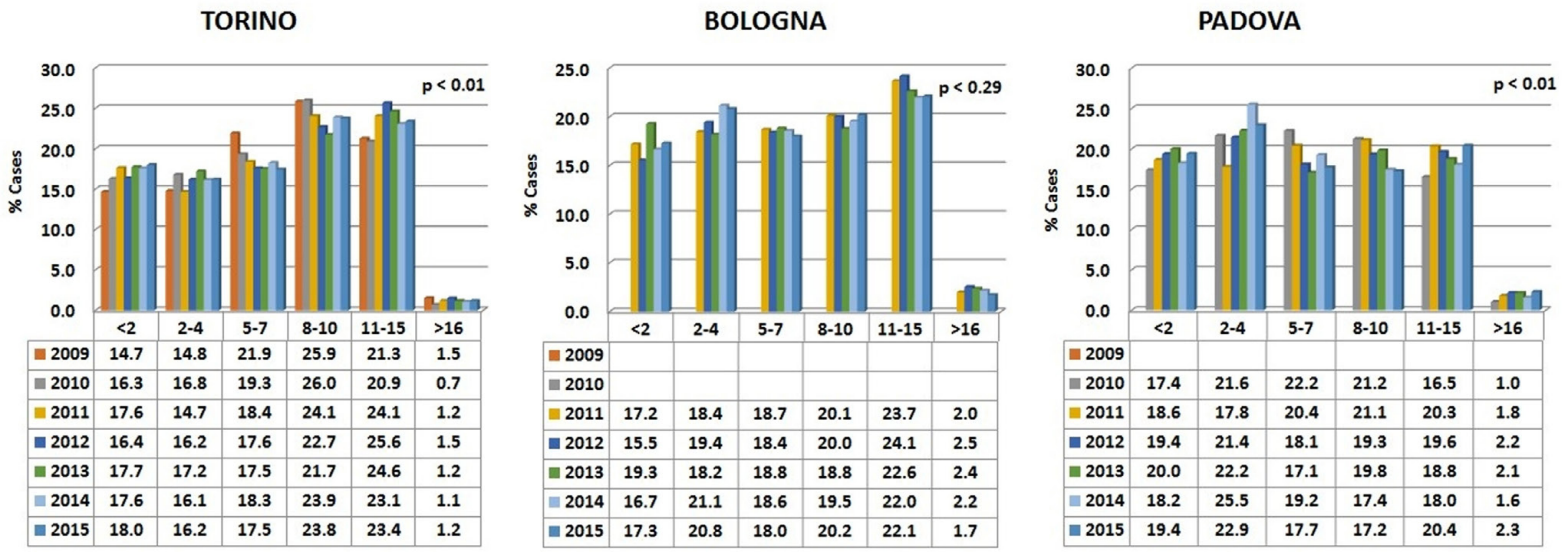
**Percentages of dogs’ visits, grouped by age classes, for each year in the three hospitals**. The probability (*p*) for the hypotheses, within each hospital, of a homogeneous distribution of different age classes in different examination years has been reported by means of a Chi-square goodness-of-fit test.

Data are reported in Figure 1. Variations among years are minimal and the percentages are relatively comparable in the different hospitals.

**Figure 2:**
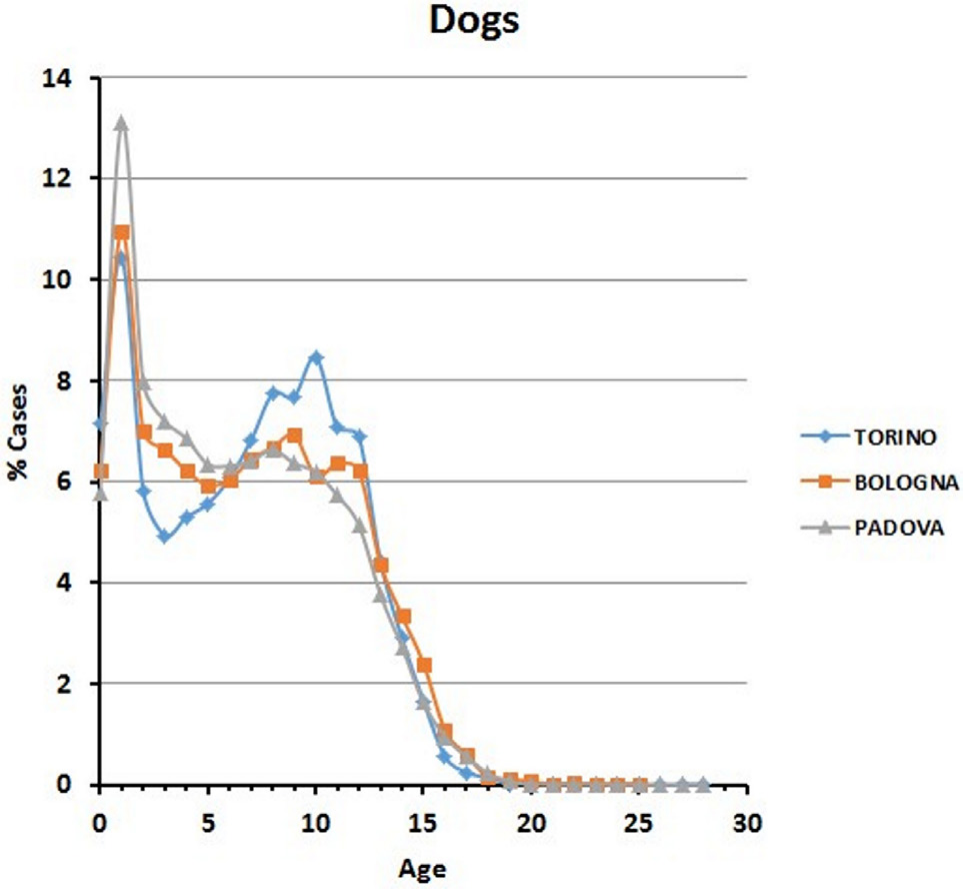
**Frequency (percentage) of dogs’ visits relative to age in the three hospitals**.

In all the three animal hospitals, the highest number of visits corresponded to the early life phases of the dogs or to middle to advanced age (Figure 2). A certain number of cases (approximately 1% in Torino, 2% in Bologna, and 2% in Padova) concerned dogs older than 16 (see also below Comparative Analysis of Data).

Table 2 reports the subdivision by sex of the dogs examined in the three hospitals. Age of the oldest visited dog in Torino was 25, in Bologna 28, and in Padova 28.

**Table 2:** **Data on the examined dogs**.

Dogs	F	M	S	C	Total
**Torino**					
Number	6,071	8,692	29,65	742	18,470
Mean age at visit ± SEM	6.2 ± 0.06	6.7 ± 0.05	8. 5 ± 0.07	9.4 ± 0.13	7.0 ± 0.03
**Padova**					
Number	7,876	9,165	3,324	1,074	21,349
Mean age at visit ± SEM	5.6 ± 0.05	6.2 ± 0.05	7.9 ± 0.07	7.8 ± 0.14	6.3 ± 0.03
**Bologna**					
Number	2,751	4,248	1,859	599	9,457
Mean age at visit ± SEM	5.8 ± 0.09	6.4 ± 0.07	8.4 ± 0.10	8.7 ± 0.17	6.8 ± 0.05
**Torino + Padova + Bologna**					
Number	16,698	22,105	8,148	2,415	49,366
Mean age at visit ± SEM	5.9 ± 0.03	6.5 ± 0.03	8.2 ± 0.05	8.5 ± 0.08	6.6 ± 0.02

*F, intact bitches; M, intact males; S, spayed bitches; C, neutered males*.

### Cats

**Figure 3:**
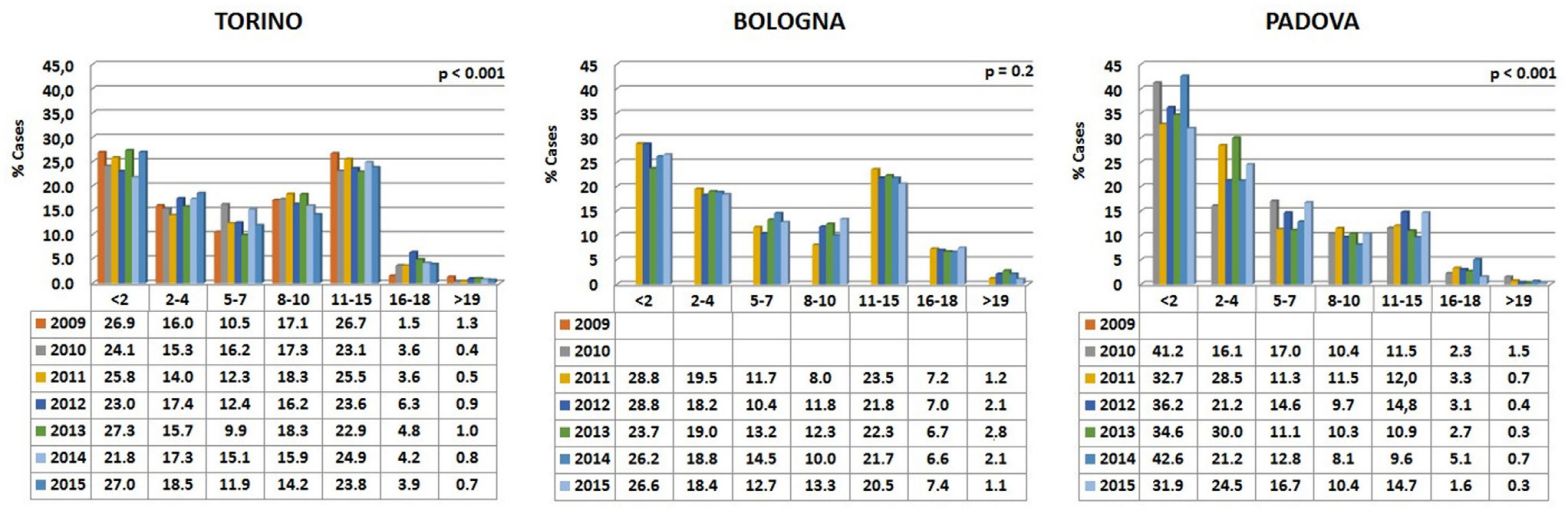
**Percentages of cats’ visits, grouped by age classes, for each year in the three hospitals**. The probability (*p*) for the hypotheses, within each hospital, of a homogeneous distribution of different age classes in different examination years has been reported by means of a Chi-square goodness-of-fit test.

**Figure 4:**
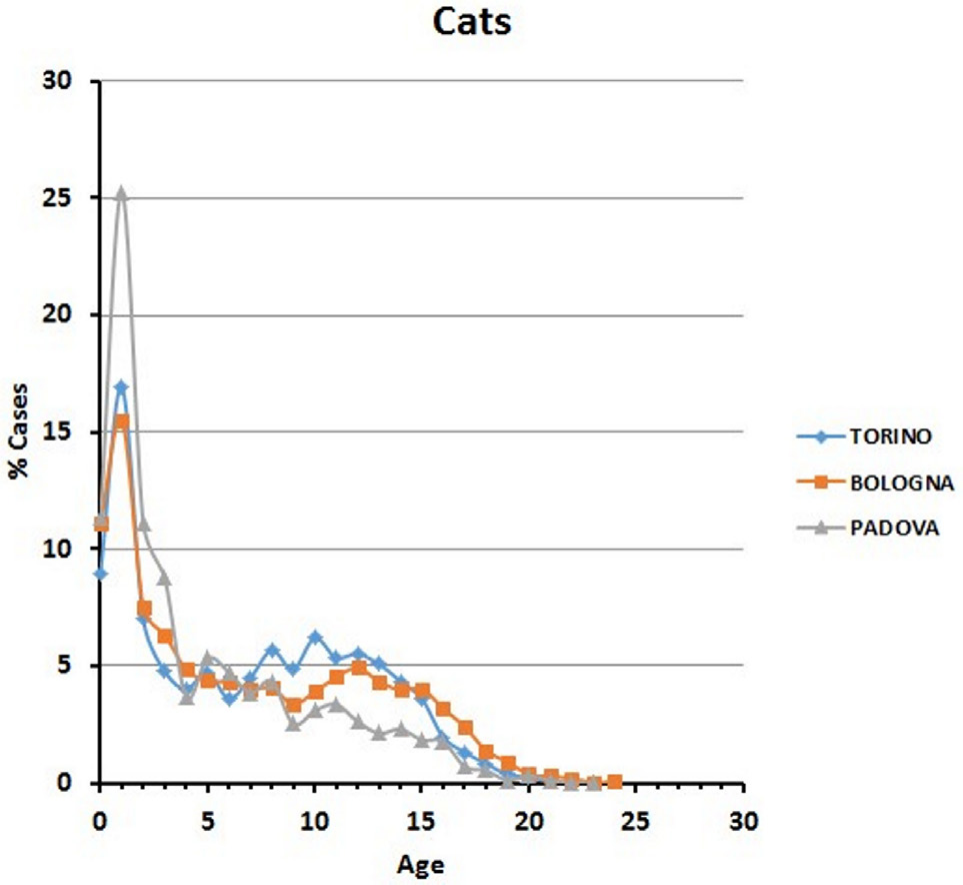
**Frequency (percentage) of cats’ visits relative to age in the three hospitals**.

Data are reported in Figure 3. Variations among years are minimal in the Bologna hospital, but more variable in the other two structures. The percentages of cats visited in the three hospitals show an initial peak relative to young animals (Figure 4). The proportion of visited cats belonging to the 11–15 age class is noticeably higher in Torino and Bologna than in Padova.

A certain number of cases (approximately 1% in Torino, 2% in Bologna, and 0.5% in Padova) concerned cats aged 19 or older (see also below Comparative Analysis of Data).

Table 3 reports the subdivision by sex of the cats examined in the three hospitals. Age of the oldest visited cats was 23 in Torino and Padova and 24 in Bologna.

**Table 3:** **Data on the examined cats**.

CATS	F	M	S	C	Total
**Torino**					
Number	994	989	1,663	1,813	5,459
Mean age at visit ± SEM	4.2 ± 0.16	4.1 ± 0.15	9.0 ± 0.12	7.8 ± 0.11	6.9 ± 0.07
**Padova**					
Number	1,092	1,170	853	827	3,942
Mean age at visit ± SEM	2.9 ± 0.12	2.8 ± 0.11	7.8 ± 0.17	7.0 ± 0.17	4.8 ± 0.08
**Bologna**					
Number	702	724	963	1,063	3,452
Mean age at visit ± SEM	3.6 ± 0.18	3.4 ± 0.17	9.6 ± 0.17	8.8 ± 0.16	6.8 ± 0.10
**Torino + Padova + Bologna**					
Number	2,788	2,883	3,479	3,703	12,853
Mean age at visit ± SEM	3.6 ± 0.09	3.4 ± 0.08	8.9 ± 0.09	7.9 ± 0.08	6.2 ± 0.05

*F, queens; M, toms; S, spayed females; C, neutered males*.

### Horses

**Figure 5:**
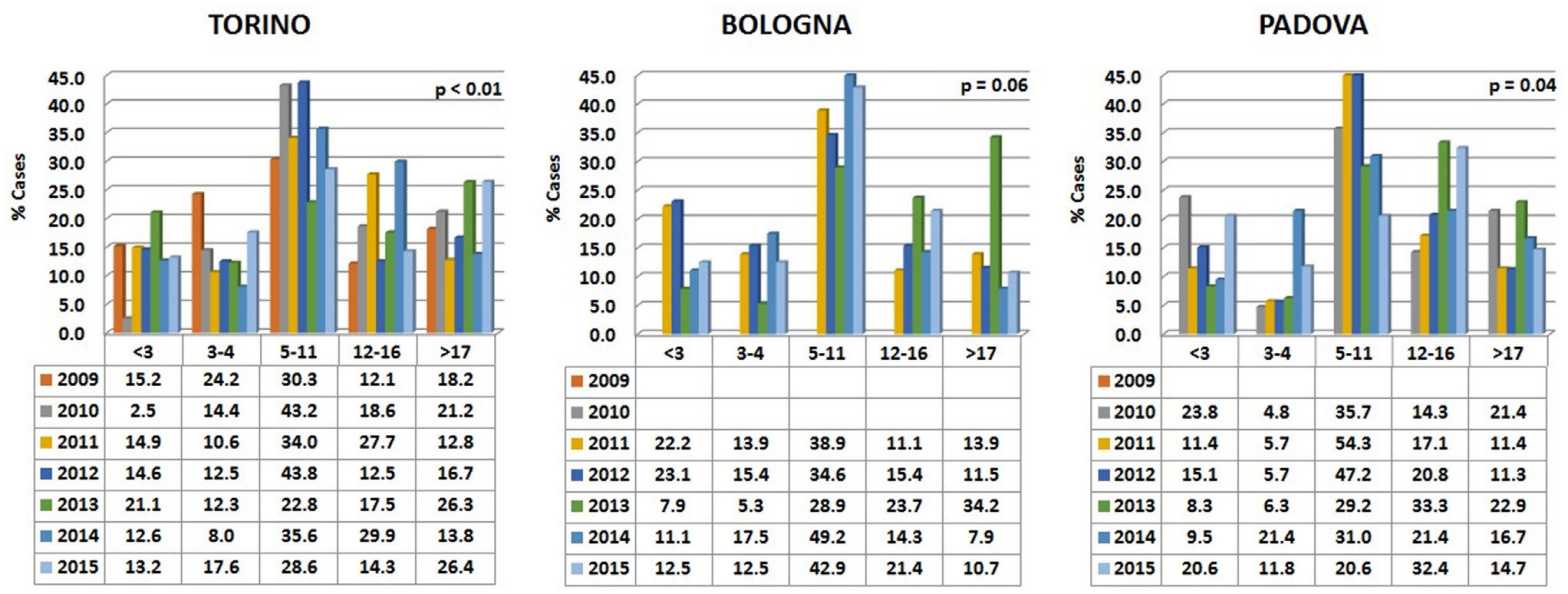
**Percentages of horses’ visits, grouped by age classes, for each year in the three hospitals**. The probability (*p*) for the hypotheses, within each hospital, of a homogeneous distribution of different age classes in different examination years has been reported by means of a Chi-square goodness-of-fit test.

Data are reported in Figure 5. Variations among years were more pronounced than in the two carnivore species, while the percentages of visited horses subdivided into age classes were relatively comparable in the different hospitals.

**Figure 6:**
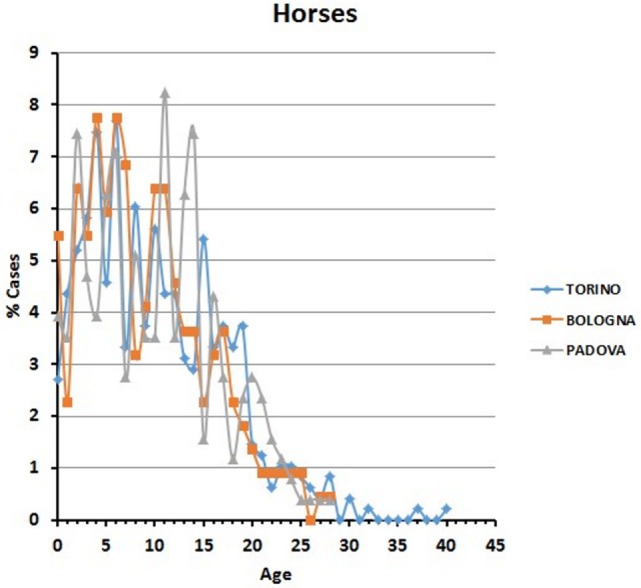
**Frequency (percentage) of horses’ visits relative to age in the three hospitals**.

In all the three hospitals, most of the visited horses were aged between 5 and 11 years (Figure 6). A certain number of cases (approximately 20% in Torino, 15% in Bologna, and 16% in Padova) concerned horses older than 17 (see also below Comparative Analysis of Data).

Table 4 reports the subdivision by sex of the horses examined in the three hospitals (the category of spayed females does not exist here, since the surgical procedure is unusual for the species). Age of the oldest visited horse was 28 in Bologna and Padova and 40 in Torino.

**Table 4:** **Data on the examined horses**.

Horses	F	M	C	Total
**Torino**				
Number	224	124	133	481
Mean age at visit ± SEM	10.8 ± 0.46	5.9 ± 0.53	13.5 ± 0.54	10.3 ± 0.32
**Padova**				
Number	104	78	72	254
Mean age at visit ± SEM	11.2 ± 0.65	7.3 ± 0.77	11.3 ± 0.59	10.0 ± 0.41
**Bologna**				
Number	103	64	52	219
Mean age at visit ± SEM	8.8 ± 0.62	7.6 ± 0.78	11.8 ± 0.78	9.2 ± 0.42
**Torino + Padova + Bologna**				
Number	431	266	257	954
Mean age at visit ± SEM	10.5 ± 0.32	6.7 ± 0.38	12.5 ± 0.37	10.0 ± 0.22

*F, mares; M, stallions; C, geldings*.

### Comparative Analysis of Data

**Figure 7:**
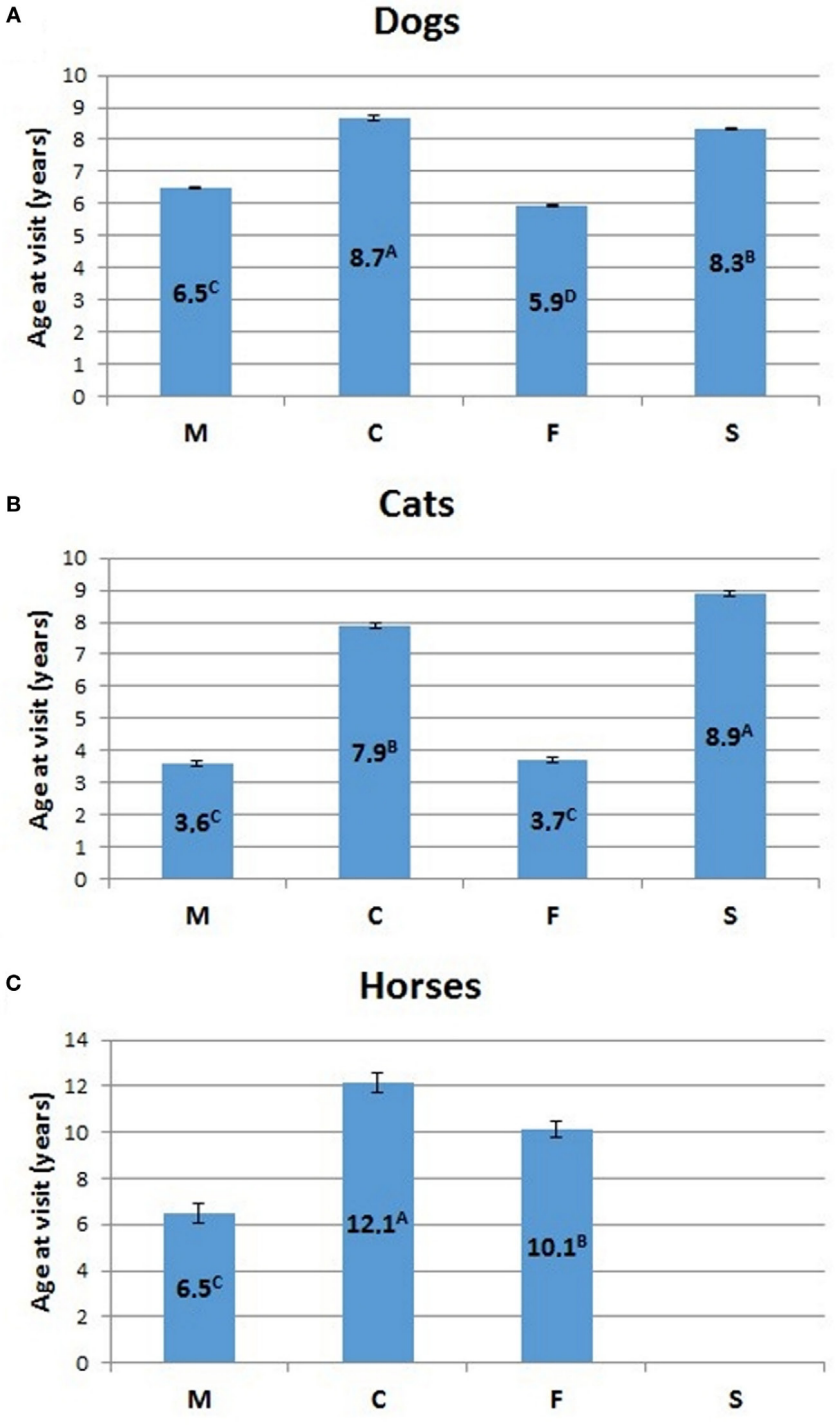
**Comparison of LSMEANS age at visit for (A) dogs, (B) cats, and (C) horses, considering intact (M, intact males; F, intact females) and neutered (C, neutered males; S, spayed females) animals**. Different letters indicate significant differences between LSMEANS at *p* < 0.01.

The relative percentage of aged animals of the three species, subdivided by sex, is summarized in Table 5. Statistical comparison of LSMEANS age at visit for dogs, cats, and horses, considering intact (M, males; F, females) and neutered (C, neutered males; S, spayed females) animals is represented in Figure 7.

**Table 5:** **Relative percentage of aged animals**.

Species	Age range	Sex		Numbers	Percentage
Cat^a^	>19	♂	Toms	9	0.39
			Neutered	41	
		♀	Queens	22	0.62
			Spayed	58	
Dog^b^	>16	♂	Intact males	285	0.70
			Neutered	61	
		♀	Bitches	245	0.93
			Spayed	215	
Horse^c^	>17	♂	Stallions	26	9.85
			Geldings	68	
		♀	Mares	76	7.97

*^a^Approximately 1% in Torino, 2% in Bologna, and 0.5% in Padova*.

*^b^Approximately 1% in Torino, 2% in Bologna, and 2% in Padova*.

*^c^Approximately 20% in Torino, 15% in Bologna, and 16% in Padova*.

The mean age at visit for dogs in our experimental cohort resulted 6.6 ± 0.02 years (mean ± SEM) (Table 2). Values for intact dogs were 5.9 ± 0.03 for females and 6.5 ± 0.03 for males, while the mean age at visit was higher for neutered dogs of both sexes (8.2 ± 0.05 for spayed females and 8.5 ± 0.08 for neutered males) (Table 2). The difference of age at visit between neutered and intact male dogs, and between intact and spayed bitches was statistically significant (*p* < 0.001) (Figure 7A).

The mean age at visit in our cohort of cats was 6.2 ± 0.05 years (mean ± SEM) (Table 3). Considering the separate categories, the mean age was 3.6 ± 0.09 for queens and 3.4 ± 0.08 for toms, but significantly (*p* < 0.001) rose to 8.9 ± 0.09 for spayed females and 7.9 ± 0.08 for neutered males (Table 3; Figure 7B).

In the three hospitals, the mean age at visit for horses was 10 ± 0.22 years (mean ± SEM) (Table 4). The age of mares (10.5 ± 0.32) was higher than that of the stallions (6.7 ± 0.38), but lower than that of geldings (12.5 ± 0.37).

**Figure 8:**
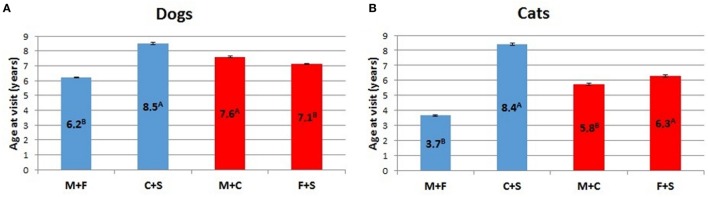
**Comparison of LSMEANS age at visit for (A) dogs and (B) cats considering intact animals (M + F) vs. neutered animals (C + S), or intact and neutered individuals of the same sex vs. individuals of the opposite sex (M + C vs. F + S) (M, intact males; F, intact females, C, neutered males; S, spayed females)**. Different letters indicate significant differences between LSMEANS at *p* < 0.01.

ANOVA analyses of potential sex differences, considering intact animals (dogs and cats) vs. neutered animals, or intact and neutered individuals of the same sex vs. individuals of the opposite sex, is represented in Figure 8. In both cases, intact cats and dogs showed a lower (*p* < 0.01) mean age than neutered individuals. For the latter category, the mean age at visit was similar in the two examined species (i.e., 8.5 for dogs and 8.4 for cats, respectively), but the average age at visit of intact dogs was almost two times that of intact cats (Figure 8). The within sex age at visit resulted greater for males than for females in dogs (*p* < 0.01), while male cats showed a lower mean age at visit than females (*p* < 0.01).

## Discussion

There is a consensus in the scientific world that there is a direct correlation between the life expectancy of a given human population and the quality of the available health care (20). Other factors potentially affect individual longevity in humans, including age of the mother at birth, place, and season of birth (21), while the role of genetic heritage in a prolonged lifespan is complex (22–27). To some extent, the same concepts apply also to rodents, although their life span is limited to few years by size and especially metabolism (28). However, data on domestic mammals are uncertain.

Our analyses of thousands of clinical cases contained in the databases of three major veterinary hospitals of Northern Italy show that treatment is indeed reserved also for old to very old animals. Young domestic carnivores are brought to the clinic for preventive routine treatments, including vaccination and antiparasite treatment (Figures 2 and 4). Neutering of domestic toms and queens generally takes place during the first year of life, thus accounting for the peak of visits of young animals. However, our data also clearly show that domestic carnivores receive qualified medical care even during their mature years (>10 and older), so much that visits to animals belonging to what we considered advanced or senior age (>16 years for dogs and >19 years for cats) were 1.6 and 1% of their respective total (Table 5). Removal of sensitive data from our files (as requested by the three hospitals) leaves us no possibility to assess whether old dogs were exclusively small dogs, as previously suggested in different studies (11, 16, 29). On the other hand, the inverse relationship between individual body mass and longevity, although demonstrated in dog breeds (30–33), is still under debate in other mammals (34). We are also aware that specific factors may be responsible for some differences in the frequency of age classes brought to visit in the three hospitals (see, for example, cats aged 11–15 years). Reasons for these discrepancies may very well include social differences or age in the population of the owners, economy of the three provinces considered, or even the density of rural vs. town settlements in the area surrounding the hospitals.

The average life span in dogs reported in the literature (see Table 6) was considered to vary between 6.7 (17) and 12 years (35), although this latter value was taken from a review and should perhaps be confirmed. Our data contain no indication of a true average life span. However, the somewhat similar parameter of the mean age at visit was 6.6 years in dogs (Table 2), a concept not necessarily corresponding to that of average life span. We also noted that the mean age at visit was higher for neutered dogs of both sexes. Neutered males and spayed bitches recorded a statistically significant (*p* < 0.001) higher age at visits than intact animals (see Figure 7). The average life span of cats (Table 6) varied between 13 and 17 years (13). The mean age at visit in our cohort of cats was 6.2 (Table 3), but the age at visit of neutered cats was significantly (*p* < 0.001) higher, with 7.9 for spayed females and 8.9 years for neutered males (Figure 7B, *p* < 0.001), possibly reflecting a higher quality of medical care reserved to pets that continued to live in the house (treatment for young animals could be directed also to stray cats living in urban colonies, subsequently seldom brought to the hospital in their older years; see also below).

**Table 6:** **Life span of selected mammalian species**.

Species	Life span (years)		Reference	Notes
	Average	Maximum		
**Domestic carnivores**				
Cat		>27	Comfort (8)	
	13–17	21	Spector (13)	
		22	Hayashidani et al. (14)	
	15	38	Grimm (35)	
		30	http://genomics.senescence.info/species	^a^
		24	Present study	

Dog	13–17	34	Spector (13)	
		>24	Comfort (12)	
	10		Eichelberg and Seine (15)	
	6.7–8.5		Patronek et al. (17)	
	12		Michell (36)	
	10		Prochowsky et al. (18)	
	12		O’Neill et al. (33)	
	12	29	Grimm (35)	
		24	http://genomics.senescence.info/species	^a^
		28	Present study	

Small- to medium-sized dogs		24	Comfort (11)	
	10	>15	Li et al. (16)	

Large-sized dogs		14	Comfort (11)	
	7	>10	Li et al. (16)	

**Domestic herbivores**				
Horse	20–30	62	Spector (13)	
	18–22	33–34	Comfort (9)	Thoroughbred ♀
	>18–22	30	Comfort (10)	Thoroughbred ♂
		>40	Comfort (12)	
		≌60	Grimm (35)	
		57	http://genomics.senescence.info/species	^a^
		40	Present study	^b^

Cattle	20–25	30	Spector (13)	
		>30	Comfort (12)	
		20	http://genomics.senescence.info/species	^a^

Sheep	10–15	20	Spector (13)	
		>15	Comfort (12)	
		22.8	http://genomics.senescence.info/species	^a^

Goat	8–10	18	Spector (13)	
		20.8	http://genomics.senescence.info/species	^a^

Rabbit		12	Comfort (12)	
	5–7	18	Cozzi et al. (37)	
		9	http://genomics.senescence.info/species	^a^

**Domestic omnivores**				
Pig	16	27	Spector (13)	
		27	http://genomics.senescence.info/species	^a^

**Primates**				
Man		>115	Comfort (12)	
	71	122	Grimm (35)	
		122	Dong et al. (6)	

Chimpanzee		25–30	Nigrelli (38)	
		>39	Comfort (12)	
		≌60	Grimm (35)	
		59.4	http://genomics.senescence.info/species	^a^

**Laboratory rodents**				
Rat		>4	Comfort (12)	
		3.8	Miller et al. (28)	
	2.5–3		Cozzi et al. (37)	
		3.8–4.2	http://genomics.senescence.info/species	^a^

Mouse		>3	Comfort (12)	
		4	Turturro et al. (39)	
	1–3		Cozzi et al. (37)	
		4	http://genomics.senescence.info/species	^a^
		4	Cohen (40)	

Guinea pig		>7	Comfort (12)	
	4–5	8	Cozzi et al. (37)	
		12	http://genomics.senescence.info/species	^a^

*^a^http://genomics.senescence.info/species is a website dedicated to genetic research in aging mammals*.

*^b^While writing the present article, we were made aware, by a practitioner related to the University of Padova, of the proven existence of a healthy gelding of 42*.

The situation is different in horses. In all the three hospitals, the majority of horses received medical attention during their young to adult age (5–11) and continued to be brought to the clinics also during their older years. Horses of advanced age (>17) continued to receive veterinary care, a further proof of the affection of the owners to animals possibly less performant, but considered true valuable pets. Here, we note that the equine population presented to visit at university veterinary hospitals may not be representative of all the companion horses, and the sample could be biased toward true “pet” horses, race horses being less represented.

For several decades, the attention on life parameters was focused on single individuals, and their maximum achievable age. Table 6 reports current data on the topic. Apparently, some old data must be taken with some caution. As an example, Mellen (41), quoted by Ref (8) reported the existence of a 31-year-old cat, based on the answers received in questionnaires distributed to animal owners. Comfort (8) considered that, in the absence of factual verification, such an age, could not be trusted. However, a recent review published in Science (35) placed the maximum life span for cats at 38 years, perhaps based on anecdotal information present in the web. The oldest cat brought to the attention of the three hospitals during the years of our study was 24, a quite considerable achievement, but definitely far from an over 30 limit. The oldest dog in our study reached 28, close to the value reported by Grimm (35), but again far from the limit of 34 previously cited (13). The oldest recorded horse lived to the age of 62 (13), but apart from that single animal, the maximum age in our study tops corresponding values reported by similar studies (see Table 6).

The effect of gender on life expectancy is relevant in humans (21, 42), and, according to the literature, it applies to dogs as well (43), with intact females having higher chances to reach advanced age. In our study, if we consider intact and sterilized animals together as both are representative of their gender, the absolute differences of age at visit between sexes are apparently minimal: however, they are significant for carnivores, possibly due to the high number of cases considered (Figures 8A,B). Neutered cats and dogs have the higher LSMEANS age (more than double for cats, Figure 8B), a result that agrees with previous studies in sterilized dogs (44). Further analyses of detailed clinical records may yield to some interesting conclusions. In all the three hospitals, intact male dogs have a mean age higher than intact bitches (see Table 2; Figure 8A), and that also applies to neutered male dogs and sterilized bitches, although these latter two categories have a higher age at visit. The situation may also derive from a gender-biased choice of the pets from the owner, as the number of male dogs is constantly higher among the patients in all the hospitals. Figure 8A suggests that, on the average, neutered (males and females) dogs have a higher age at visit than intact animals considered together. Our data have no specific background that qualifies and categorizes the single clinical visits, so that we cannot state whether gonadectomy is associated with the prevision of longer age. Quite possibly, the present results are indicative of the tendency of the owners to house sterilized pets for their docility.

Neutered cats have a considerably higher mean age than toms and queens (Table 3; Figure 8B), a factor that possibly reflects the diffusion of early gonadectomy in this species as a prerequisite for domestic life. In fact, the number of male and female cats brought to the clinics is equivalent. A further contributing factor is that several cats live their full life within doors or in a confined environment.

Differences of age at visit are complex to consider for horses, since mares apparently are older than stallions, but less than geldings (Table 4; Figure 7C). The LSMEANS age at visit of mares is higher than that of the stallions, but geldings have an even higher LSMEANS age. In fact the LSMEANS age of intact animals (stallions and mares together) is lower than that of geldings. This latter is just an indication of the diffusion of castration as a necessary measure to maintain male horse as companion animals. Recent postmortem studied performed in mature (15–19 years) and aged equids (>20 years) showed that both genders were equally represented (45).

Our study suggests that a considerable number of dogs, cats, and horses attain an advanced age as defined by the categories that we adopted to subdivide our experimental cohort. The data, based on hospital admissions, indicate that the present tendency in veterinary medical practice allows a certain number of female carnivores and mares to age (see Table 5). It is generally accepted that females of domestic mammals show a decline in ovarian activity and an increase in reproductive pathologies during aging, but absolute limits of reproductive capabilities are not clear as naturally occurring sexual activity and mating are strictly controlled or prevented in aged animals for either medical or practical reasons, including decreasing-to-disappearing of fertility rates in mares. It is well known that only few species (humans, chimpanzees, lemurs, killer, and pilot whales) have an extended post-reproductive lifespan. According to Cohen (46), the definition of menopause corresponds to the irreversible loss of the physiological capacity to produce offspring due to intrinsic biological factors, while reproductive senescence is the decline in fecundity after its peak. Only a few non-human mammalian species attain a menopause (47–51). The phenomenon has a specific evolutionary advantage since it allows expert females to participate in rearing of the young ones and even contribute to transmission of social habits and group strategies (51) in species with high degree of sociality.

The value of the oldest records achieved for the different species has a limited practical value, perhaps just implying a possible goal for the animal owner. On the other hand, the data that we collected and report here suggest that veterinary attention is consistently dedicated to a robust percentage of mature to old pets and, potentially, really old horses. In our opinion, the growing consideration for old companion animals testifies an evolution in the animal–owner relationship and a renewed respect for the value of life in companion domestic mammals.

## Author Contributions

BC and AR conceived the research, obtained the data, examined the results, and wrote the article. CB elaborated the data. RM performed the statistical analyses.

## Conflict of Interest Statement

The authors declare that the research was conducted in the absence of any commercial or financial relationships that could be construed as a potential conflict of interest.

## Supplementary Material

Data Sheet 1 **Extended datasheet from the three University Veterinary Hospitals**.
